# Enhanced engraftment, proliferation, and therapeutic potential in heart using optimized human iPSC-derived cardiomyocytes

**DOI:** 10.1038/srep19111

**Published:** 2016-01-08

**Authors:** Shunsuke Funakoshi, Kenji Miki, Tadashi Takaki, Chikako Okubo, Takeshi Hatani, Kazuhisa Chonabayashi, Misato Nishikawa, Ikue Takei, Akiko Oishi, Megumi Narita, Masahiko Hoshijima, Takeshi Kimura, Shinya Yamanaka, Yoshinori Yoshida

**Affiliations:** 1 Center for iPS Cell Research and Application, Kyoto University, Kyoto, Japan; 2 Department of Cardiovascular Medicine, Kyoto University Hospital, Kyoto, Japan; 3 Center for Research in Biological Systems and Department of Medicine, University of California at San Diego, La Jolla, California, USA; 4 Gladstone Institute of Cardiovascular Disease, San Francisco, USA

## Abstract

Human pluripotent stem cell-derived cardiomyocytes (CMs) are a promising tool for cardiac cell therapy. Although transplantation of induced pluripotent stem cell (iPSC)-derived CMs have been reported in several animal models, the treatment effect was limited, probably due to poor optimization of the injected cells. To optimize graft cells for cardiac reconstruction, we compared the engraftment efficiency of intramyocardially-injected undifferentiated-iPSCs, day4 mesodermal cells, and day8, day20, and day30 purified iPSC-CMs after initial differentiation by tracing the engraftment ratio (ER) using *in vivo* bioluminescence imaging. This analysis revealed the ER of day20 CMs was significantly higher compared to other cells. Transplantation of day20 CMs into the infarcted hearts of immunodeficient mice showed good engraftment, and echocardiography showed significant functional improvement by cell therapy. Moreover, the imaging signal and ratio of Ki67-positive CMs at 3 months post injection indicated engrafted CMs proliferated in the host heart. Although this graft growth reached a plateau at 3 months, histological analysis confirmed progressive maturation from 3 to 6 months. These results suggested that day20 CMs had very high engraftment, proliferation, and therapeutic potential in host mouse hearts. They also demonstrate this model can be used to track the fate of transplanted cells over a long time.

Despite the vast improvements in heart failure prognosis, treatment efficiency is significantly limited for patients with severely decreased cardiac function. Consequently, in many cases, cardiac transplantation is often the only treatment option, however, there is a chronic shortage of donor hearts^1^. A therapeutic alternative to heart transplantation is thus required^2^. Cardiac cell therapy is one such promising strategy. In the past decade, several stem cell therapies, such as bone marrow progenitors and cardiac stem cells, have been explored in the clinical setting^3^,^4^,^5^. Unfortunately, their treatment effects are limited, probably because the effects depend mainly on paracrine effects by the transplanted cells and not on the recovery of the number of functioning cardiomyocytes (CMs). To reconstruct the myocardium and improve the treatment effect of cell therapy, an efficient method for the transplantation and engraftment of CMs themselves is desired.

Human pluripotent stem cells (PSCs), including embryonic stem cells (ESCs) and induced pluripotent stem cells (iPSCs), which have the ability to proliferate without limit and differentiate into several cell types^6^,^7^, are expected to be sources for cardiac cell therapy^8^ and have been explored for this purpose in experimental models. Already, several studies have reported that the transplantation of PSC-derived cardiomyocytes into damaged hearts improves cardiac function^9^,^10^,^11^. However, poor engraftment capacity shows that considerable improvement in this method is needed.

One reason is that the injected cells are not optimal^12^,^13^. It is possible that dynamic changes in the cellular phenotypes during the differentiation of PSCs into CMs affect the outcome^14^,^15^. Therefore, there may exist an optimal differentiation stage for cardiac cell therapy. In the present study, we compared the engraftment ratio (ER) of CMs at different stages of differentiation using bioluminescence imaging and elucidated that iPSC-CMs 20 days (day20 CMs) after the initial differentiation had the highest engraftment capacity. When day20 CMs were injected into the infarcted hearts of immune-deficient mice, significant improvement in function was observed, suggesting the therapeutic potential of these cells. Moreover, to better understand the behavior of the injected cells, we observed phenotypic changes, including proliferation and maturation, for 6 months, which is a period much longer than observed in previous reports.

## Results

### Cardiac differentiation and characteristics of iPSC-derived cardiomyocytes

**Figure 1:**
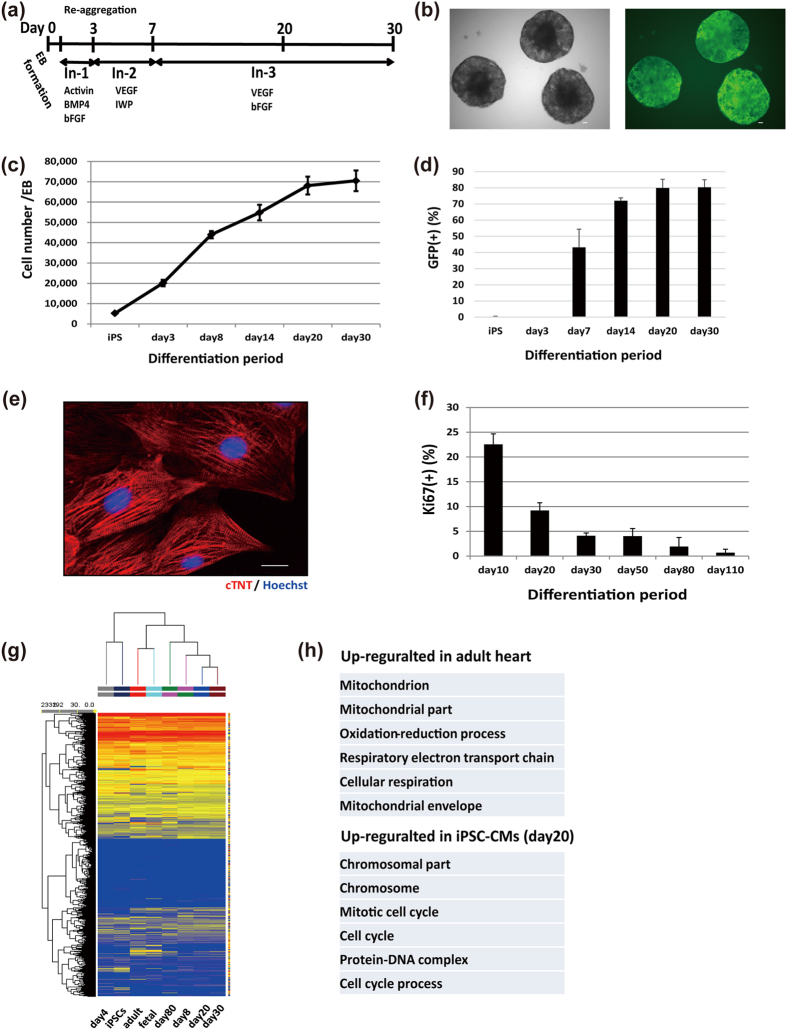
Cardiac differentiation and characteristics of iPSC-derived cardiomyocytes. (**a**) Schematic representation of the cardiac differentiation protocol. (**b**) Representative images of differentiated embryoid bodies. Left: bright-field; right: GFP. Scale bars: 100 μm. (**c**) Increasing number of cells per embryoid body during cardiac differentiation (n = 3). (**d**) Percentage of GFP-positive cells during differentiation (n = 3).(**e**) Immunostaining on day 20 of purified CMs: red, cTNT; blue, Hoechst. Scale bar: 20 μm. (**f**) Percentage of Ki67-positive CMs *in vitro* (n = 3) over different days. Data are represented as mean ± SD. (**g**) Hierarchical clustering of the global gene expression data obtained from undifferentiated iPSCs, day4 mesodermal cells, adult and fetal human hearts, and purified day8, 20, 30, and 80 iPSC-CMs. (**h**) Gene ontology analysis of differentially expressed genes between day20 iPSC-CMs and adult heart (fold change > 2.0, p < 0.05).

We used a cardiomyocyte-specific EGFP reporter human iPSC line (MYH6-EIP4) and confirmed the differentiation of iPSCs into MYH6-GFP-positive CMs using a cardiac differentiation protocol (Fig. 1a,b). The cell number increased rapidly during the first two weeks (Fig. 1c). GFP-positive CMs began to appear at 7 days, and the differentiation efficiency was approximately 80% at day 20 and day 30 after the differentiation induction (Fig. 1d and Supplementary Fig. S1a online). By sorting the GFP-positive cells, we obtained CMs with a purity of ~97% from the differentiated population on day 20 (Supplementary Fig. S1b online), and purified CMs 20 days after the initial differentiation showed clearly organized sarcomere structures (Fig. 1e). We compared changes in the gene expression profiles during the differentiation process using microarray analysis after purifying the CMs. Day4 mesodermal cells expressed mesodermal genes, such as T and MESP1/2. On the other hand, cells 8 days after the initial differentiation expressed cardiac specific genes such as MYH6 and cTNT. Between days 8 and 80, the expression of sarcomeric genes, such as MYL2, MYH7, TCAP, and MYOM2, had gradually increased to levels that approximated those seen in fetal heart samples. While the expression levels of some genes related to excitation contraction-coupling, such as KCNH2 and CACNA1C, immediately increased to levels similar to those of fetal and adult heart samples, the expression levels of RYR2 and KCNJ2 were relatively low compared to fetal and adult heart samples, although they gradually increased during long-term culture (Supplementary Fig. S2 online). The number of differentiated cells increased during the first two weeks, whereas neither day20 nor day30 iPSC-CMs increased in cell number *in vitro* (Fig. 1c). In addition, we observed the ratio of Ki67-positive CMs to be more than 20% by day 10, but to continuously decrease until hardly any Ki67-positive CMs were observed after long-term culture (Fig. 1f). Using microarray analysis, we compared the global gene expression profiles of iPSC-CMs and adult CMs. Clustering analysis revealed that iPSC-CMs and adult heart samples belong to different clusters (Fig. 1g). According to gene ontology analysis, cell cycle- and mitosis-related genes were enriched in the up-regulated genes of day20 iPSC-CMs compared to adult CMs, while mitochondria-related genes were enriched in down-regulated genes (Fig. 1h). These findings raised the possibility that iPSC-CMs had proliferation potential even though day20 CMs hardly increased in number *in vitro*.

### iPSC-CMs 20 days after initial differentiation are optimal for injection

**Figure 2:**
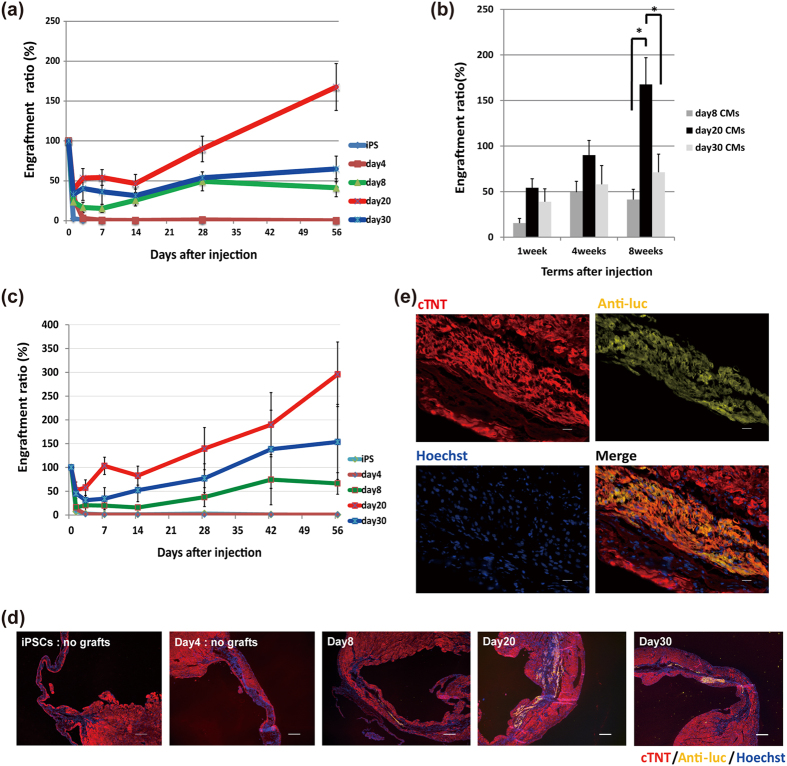
Engraftment capacity during different cardiac differentiation stages. (**a**) Engraftment ratio in the first 2 months after injection into healthy heart. iPSCs and day4 mesodermal cells, n = 5 each; day8, 20, and 30 iPSC-CMs, n = 7 each. Values are mean ± SE. (**b**) Engraftment ratio of purified CMs (day8, 20, and 30, n = 7 each) at 1, 4, and 8 weeks after the initial injection. Values are mean ± SE. *p < 0.05 by one-way ANOVA followed by Tukey’s posthoc test. (**c**) Engraftment ratio in the first 2 months after injection into infarcted heart. iPSCs, day4 mesodermal cells, and day8, 20, and 30 iPSC-CMs, n = 4 each. Values are mean ± SE. (**d**) Immunostaining of engrafted CMs in infarcted heart at each differentiation stage for cTNT (red), anti-luciferase (luc) (yellow), and Hoechst (blue). Scale bars: 300 μm. (**e**) Immunostaining of engrafted CMs in infarcted hearts at high magnification: red, cTNT; yellow, anti-luc; blue, Hoechst. Scale bars: 20 μm.

Because the phenotype of iPSC-CMs, including gene expression, changed dynamically during cardiac differentiation, we investigated the relationship between the iPSC-CMs differentiation level and the engraftment capacity. To identify the optimal differentiation stage for cardiac cell therapy, we compared the engraftment capacity among undifferentiated iPSCs, day4 mesodermal cells, and purified day8, day20 and day30 iPSC-CMs. We first injected 2.5 × 10^5^ cells of each cell type into the healthy hearts of immunodeficient interleukin-2 receptor γ^null^ (NOD/Shi-scid Il2rg^null^) (NOG) mice^16^ and measured the ER, which was defined as the percentage of the luminescence signal of the remaining grafts at a given time divided by the signal just after the graft injection^17^,^18^,^19^. We confirmed that the luminescence signal correlated with the cell number (Supplementary Fig. S3a online) and that it did not change with cell differentiation (Supplementary Fig. S3b online). When undifferentiated iPSCs and day4 mesodermal cells were injected (n = 5 each), the luminescence signal decreased rapidly and became undetectable within the first week (Fig. 2a). During a 2-month follow-up, we observed neither an increase in the luminescence signal nor teratoma formation. On the contrary, after injection of day8, day20, and day30 CMs (n = 7 each), the luminescence signal decreased during the first few days, but gradually increased afterward. Detailed comparisons of the three types of CMs found the signals of day20 and day30 CMs tended to be relatively higher than that of day8 CMs at 1 week (day8 CMs: 15.4 ± 5.1%; day20 CMs: 54.2 ± 9.9%; day30 CMs: 38.9 ± 14.1%). After 2 weeks from the initial injection, the signal of the day20 CMs increased and achieved a significantly higher signal at 2 months compared to the other CMs (day8 CMs: 41.3 ± 11.2%; day20 CMs: 167.5 ± 29.3%; day30 CMs: 71.2 ± 20.0%; p < 0.05) (Fig. 2b). Because of the high correlation between the luminescence signal and cell number, the increased luminescence signal during the observation period implied the possibility of an increased number of engrafted cells. When sorted GFP-negative non-CMs were injected, the luminescence signal showed no increase during 2 months, ruling out the possibility that residual non-CMs proliferated *in vivo* (Supplementary Fig. S4 online).

We next confirmed whether this optimization can be applied to hearts with myocardial ischemia. We injected 2.5 × 10^5^ cells of each cell type into NOG mouse heart just after ligation of the left anterior descending coronary artery and measured the ER. With similar tendency to injection into healthy hearts, the ER of day20 CMs was higher compared to other cell types (Fig. 2c,d). Additionally, histological analysis showed that the engrafted cells were cardiomyocytes stained by antibody against cardiac isoform of troponin T (cTNT) (Fig. 2d,e). These results validated day20 CMs as the optimal cell type for engraftment. Clustering analysis of the expression levels of cell adhesion genes obtained by microarray data revealed that iPSCs and day4 cells were located in clusters distinct from those of iPSC-CMs (Supplementary Fig. S5 online). Furthermore, middle-stage iPSC-CMs (day20 and day30 CMs) and early ones (day8 CMs) were in different clusters, suggesting that middle-stage and early iPSC-CMs had different characteristics regarding the expression of cell adhesion genes (Supplementary Fig. S5 online).

### Day20 CMs had therapeutic effects on damaged heart with massive engraftment

**Figure 3:**
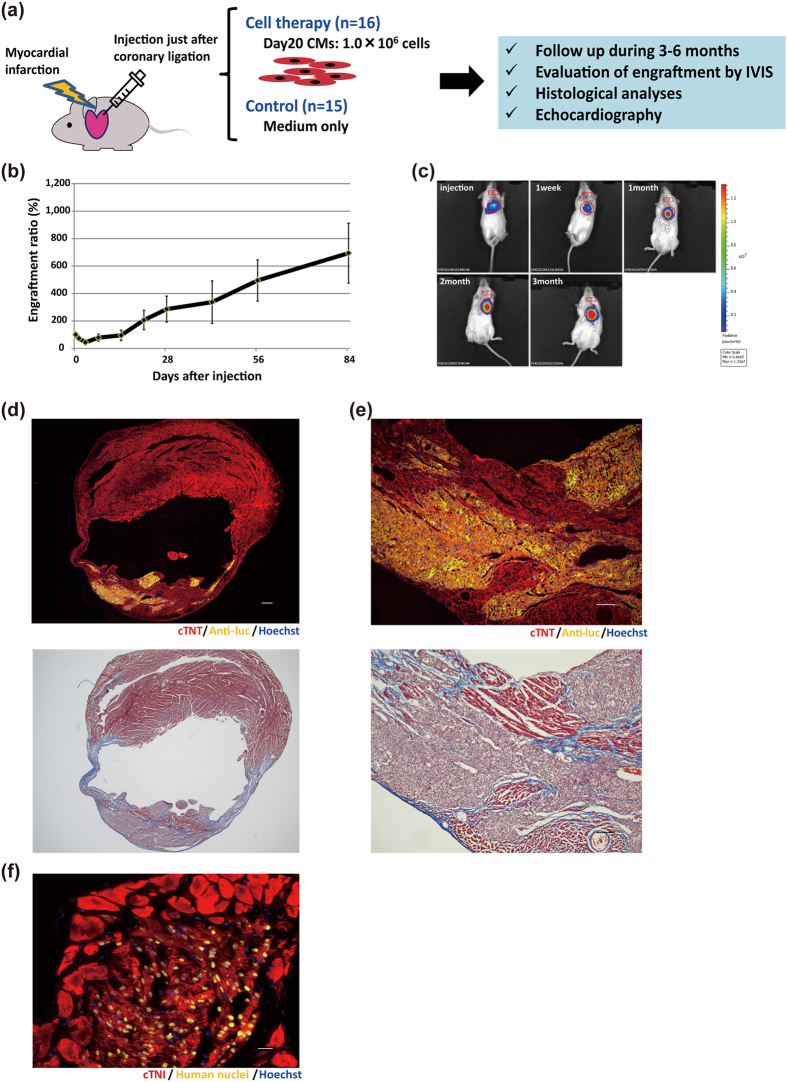
Engraftment of iPSC-CMs in infarcted mouse hearts. (**a**) Schematic summary of cell therapy using day20 CMs. (**b**) Engraftment ratio after injection of day20 iPSC-CMs based on luminescence imaging data (n = 16). Values are mean ± SE. (**c**) Representative images of luminescence signals after injection into the hearts of NOG mice with myocardial infarction. (**d**) Upper: Immunostaining of engrafted CMs in the whole heart for cTNT (red), anti-luc (yellow), and Hoechst (blue). Scale bar: 300 μm. Images were photographed by a fluorescence microscope, BZ-X700 (Keyence). Lower: Trichrome-staining of the additional section of the upper image. (**e**) Upper: Magnified image of (**d**). Red, cTNT; yellow, anti-luc; blue, Hoechst. Scale bar: 100 μm. Lower: Trichrome-staining of the additional section of the upper image. (**f**) Immunostaining of engrafted CMs at high magnification. Red, cTNI; yellow, human nuclei; blue, Hoechst. Scale bar: 20 μm.

To confirm whether our iPSC-CM based cell therapy has a therapeutic effect, we performed serial echocardiography measurements in NOG mice with myocardial infarction that were treated with iPSC-CMs (n = 16) and compared them to mice treated with medium alone (n = 15) (Fig. 3a). Because graft size was expected to increase depending on the number of injected cells^20^, we injected a larger number of purified day20 CMs (1.0 × 10^6^ cells) into NOG mouse heart with myocardial infarction. The luminescence signal decreased to 44.5% the initial signal during the first 3 days, but then increased gradually during the following 3 months, reaching about 7 times the initial intensity (Fig. 3b,c). Histological analysis at 3 months showed large amounts of engrafted CMs (Fig. 3d,e). Trichrome staining marked fibrotic areas and highlighted that a significant amount of injected CMs had engrafted in the infarcted areas and that a part of host CMs survived around the graft (Fig. 3d,e). We confirmed that most of the human nuclei-positive engrafted cells were positive for several cardiac markers, including cardiac isoform of troponin I (cTNI; TNNI3) and human NKX2-5 (Fig. 3f and Supplementary Fig. S6 online), indicating that the engrafted cells were mostly human CMs. Moreover, the engrafted CMs had clearly organized sarcomere structures (Fig. 3f). Furthermore, we also confirmed the expression of Connexin 43 at the interface of engrafted CMs and adjacent host CMs (Supplementary Fig. S7 online), although most of the engrafted areas were surrounded by modest interface scar tissue (Fig. 3e).

**Figure 4:**
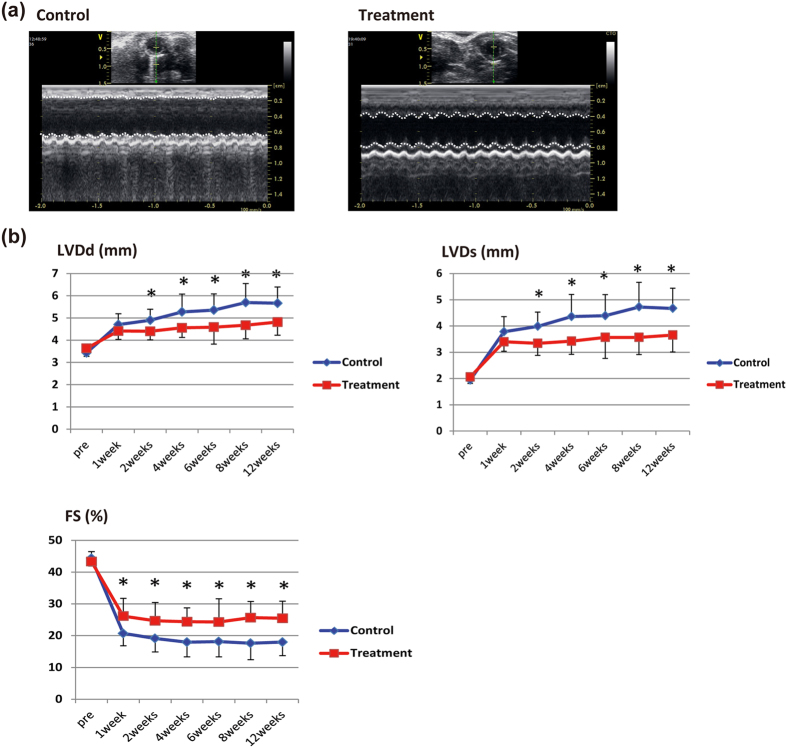
Therapeutic effect of engrafted iPSC-CMs. (**a**) Echocardiographic findings at 3 months. Left: control mouse; right: cell-treated mouse. Dotted lines: boundary of anterior and posterior walls of left ventricle. (**b**) Echocardiographic parameters (LVDd, LVDs, and FS). Values are mean ± SD. *p < 0.05 by the unpaired t test.

Echocardiography showed that control mice had typical left ventricle (LV) remodeling with increased LV dimensions and decreased fractional shortening (FS) after the myocardial infarction (Fig. 4). On the other hand, the decrease in LV function was mild in iPSC-CM treated mice. LV dimensions (LVDd and LVDs) were rapidly increased just after infarction in both mice, and after 1 week there was no significant difference between the two groups. However, while LV dilatation gradually progressed in control mice, it remained small in treated mice. As a result, LVDd and LVDs were significantly lower in the treated mice 2 weeks after the initial injection. Likewise, FS was preserved better in treated mice. At 1 week after injection, FS was significantly higher in treated mice than in control mice (26.1 ± 5.6% vs. 20.7 ± 3.9%; p < 0.01, mean ± SD), and the difference between the two increasingly widened up to 3 months (25.4 ± 5.4% vs. 17.9 ± 4.3%; p < 0.001, mean ± SD) (Fig. 4). The echocardiographic parameters were thus significantly better in the treated mice, further supporting the therapeutic potential of iPSC-CMs in cardiac cell therapy.

### Long-term follow-up revealed long-term engraftment and cardiac maturation in infarcted hearts

**Figure 5:**
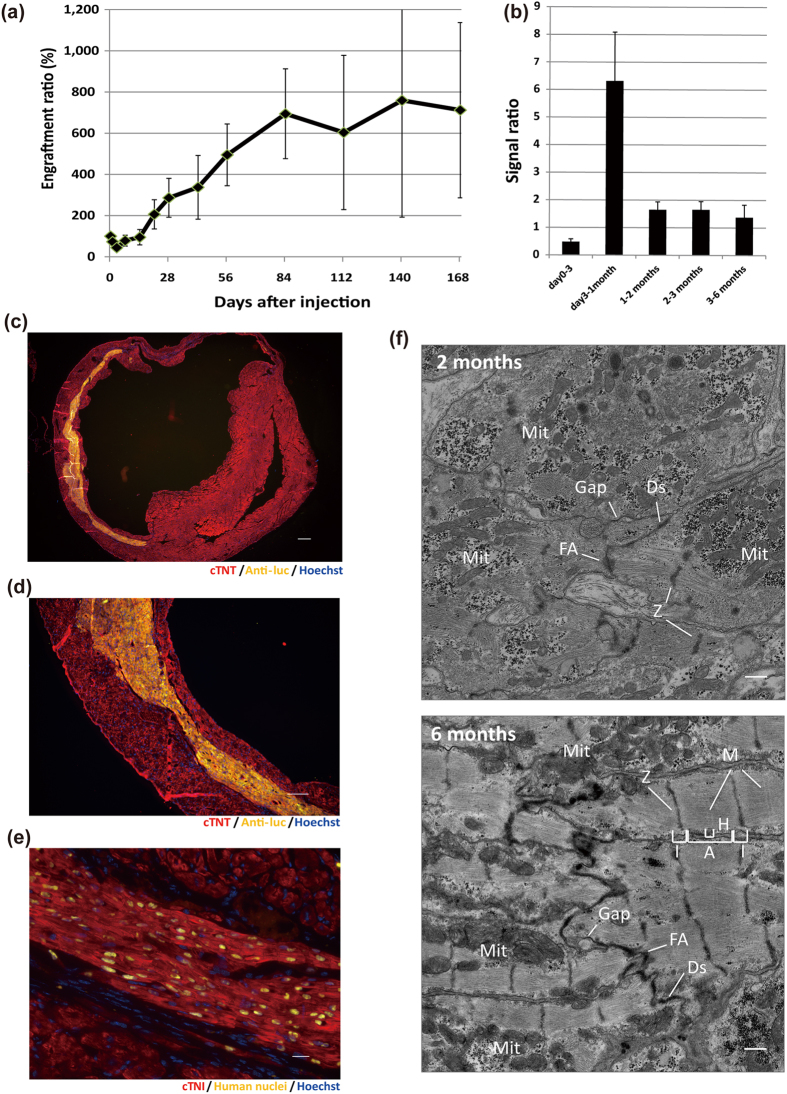
Follow-up analysis showed efficient engraftment over a long time and maturation of engrafted CMs *in vivo* in infarcted hearts. (**a**) Engraftment ratio over 6 months after injection of day20 CMs based on luminescence imaging data (day0–3 months: n = 16; 3 months-6 months: n = 5). Values are mean ± SE. (**b**) The luminescence signal ratio at several time points. The ratio was defined as the luminescence signal intensity at the end of the time interval divided by the intensity at the beginning of the interval. Values are mean ± SE.(**c**) Immunostaining of engrafted CMs in the whole heart at 6 months: red, cTNT; yellow, anti-luc; blue, Hoechst. Scale bar: 300 μm. Images were photographed by a fluorescence microscope BZ-X700 (Keyence). (**d**) Magnified image of (**c**): red, cTNT; yellow, anti-luc; blue, Hoechst. Scale bar: 100 μm. (**e**) Immunostaining of engrafted CMs at high magnification: red, cTNI; yellow, human nuclei; blue, Hoechst. Scale bar: 20 μm. (**f**) Transmission electron micrographs of iPSC-CMs at 2 months and 6 months after transplantation. Z, Z-disk; M, M-band; I, I-band; H, H-band; A, A-band; Mit, Mitochondrion; FA, Fascia Adherens; Ds, Desmosome; Gap, Gap junction. Scale bars: 500 nm.

Because the luminescence signal gradually increased over 3 months after the initial injection, we randomly selected and continued the observation of 5 treated mice with myocardial infarction to explore whether the signal continued to increase for much longer periods of time post injection. Unlike the first 3 months, the signal was relatively constant between 3 and 6 months (Fig. 5a). We also found that the signal ratio peaked between day 3 and 1 month after the initial injection, increasing 6 fold at the end of this period (Fig. 5b). On the other hand, the ratio between 1 and 3 months and 3 and 6 months was about 1.5 and 1.0, respectively (Fig. 5b). These results suggested that transplanted CM grafts did not continue to grow *in vivo*. Histological analysis, including correlative light and electron microscopic imaging, showed large amounts of engrafted CMs with increasingly organized sarcomere structures, gradually increased cTNI expression, maturation of mitochondria and inter-cellular junctions, and hypertrophic changes over time (Fig. 5c–f and Supplementary Figs S8, S9, S10 online). At 6 months, EM found that the density of myofilaments was higher and the myofilaments were longitudinally more orderly aligned with tightly organized Z-disks compared to those in 2-months cells. M-bands also started to appear in 6-months cells, in addition to A-, I-, and H-bands. The size of individual mitochondria increased and cristae became enriched. The fascia adherences, desmosomes, and gap junctions, which are three structural components constituting intercalated discs, became more discernible over the time of observation (Fig. 5f). These findings indicate sarcomeric maturation over a long period.

### Engrafted iPSC-CMs can proliferate *in vivo* 3 months after injection in infarcted hearts

**Figure 6:**
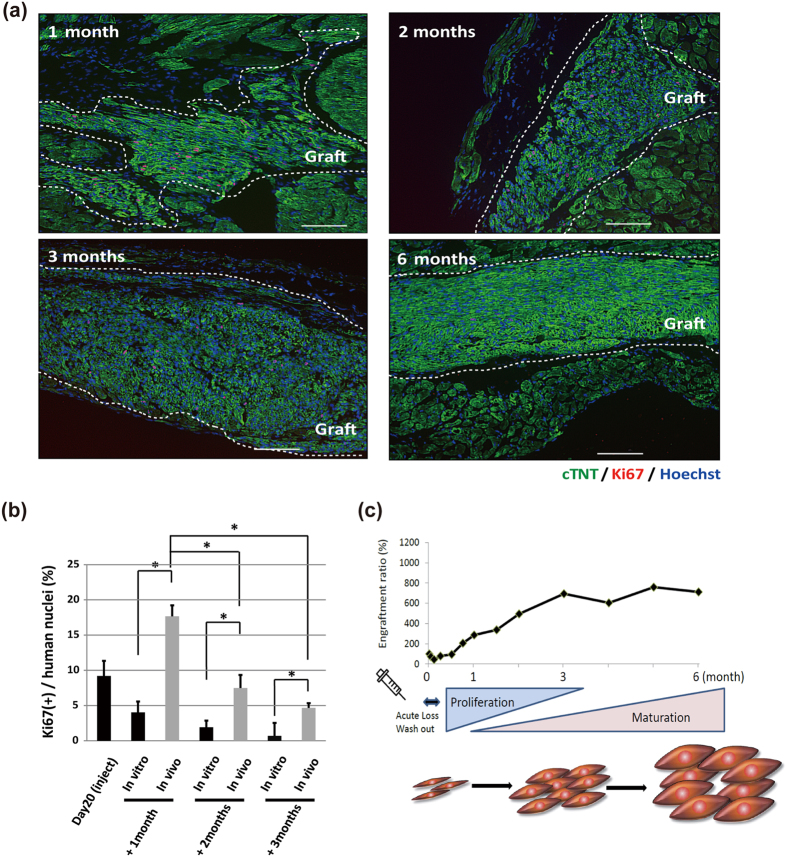
Proliferation capacity of iPSC-CMs *in vivo* in infarcted mouse hearts. (**a**) Comparison of Ki67 immunostaining of engrafted CMs at several time points: green, cTNT; red, Ki67; blue, Hoechst. Grafted areas are enclosed by white dotted lines. Scale bars: 100 μm. (**b**) Percentage of Ki67-positive CMs at several time points. Values are mean ± SD. *p < 0.05 by one-way ANOVA followed by Tukey’s posthoc test. (**c**) Schematic summary of the luminescence signal time course shown in Fig. 5a after direct injection of day20 CMs.

The luminescence signal of transplanted day20 iPSC-CMs decreased within the first 3 days of injection but gradually increased up to 3 months later (Fig. 5a). The ratio over time was highest between day 3 and 1 month and gradually decreased thereafter, not changing between 3 and 6 months. To confirm whether the engrafted CMs proliferated *in vivo*, engrafted CMs were immunostained using an antibody against the proliferation marker Ki67. We calculated the percentage of Ki67-positive CMs by the number of Ki67-positive nuclei divided by the total number of human nuclei (Fig. 6a and Supplementary Fig. S11 online) and compared the percentage of Ki67-positive CMs *in vitro* (Fig. 1f). A relatively higher ratio of Ki67-positive engrafted CMs was observed at 1 month, but only a few Ki67-positive CMs were observed at 6 months (Fig. 6a). Furthermore, the ratio at 1 month was significantly higher than that at 2 or 3 months. Moreover, we found the ratio was significantly higher in engrafted CMs than that in cultured CMs at the same time points (Fig. 6b). Again, only a small number of Ki67-positive CMs were detected at 6 months *in vivo*, which is consistent with the result of the luminescence signal. These results suggested that about half of the injected iPSC-CMs survived in damaged heart and proliferated the first 3 months after engraftment. In addition, based on the signal intensity, the engrafted CM grafts grew about 7 fold, especially during the first month after engraftment, but gradually less so with time.

As shown in Fig. 1h, a major difference in gene expressions between iPSC-CMs and adult CMs was the expression pattern of cell cycle-related genes. When we compared the expressions of these genes, we confirmed that gene expression profiles of day8–30 iPSC-CMs were closer to that of fetal heart than that of adult heart (Supplementary Fig. S12a online). Moreover, cell cycle-related genes in iPSC-CMs were more activated than those in adult CMs, although less so than in undifferentiated iPSCs (Supplementary Fig. S12b online). Furthermore, the ratio of multinucleated CMs in day20 and day30 CMs was less than 5% (Supplementary Fig. S13a,b online), which is quite lower than that in matured adult CMs^21^,^22^, and the remainder of these CMs were mononucleated. These analyses indicated that iPSC-CMs have proliferation potential and proliferated in NOG mouse heart after engraftment despite hardly proliferating *in vitro*.

In summary, the engrafted iPSC-CMs survived at least 6 months after injection and proliferated well during the first month, but gradually lost their proliferation capacity, which resulted in mature and non-proliferative CMs at 6 months (Fig. 6c).

### Optimized transplantation is applicable to different cell lines and selection methods

**Figure 7:**
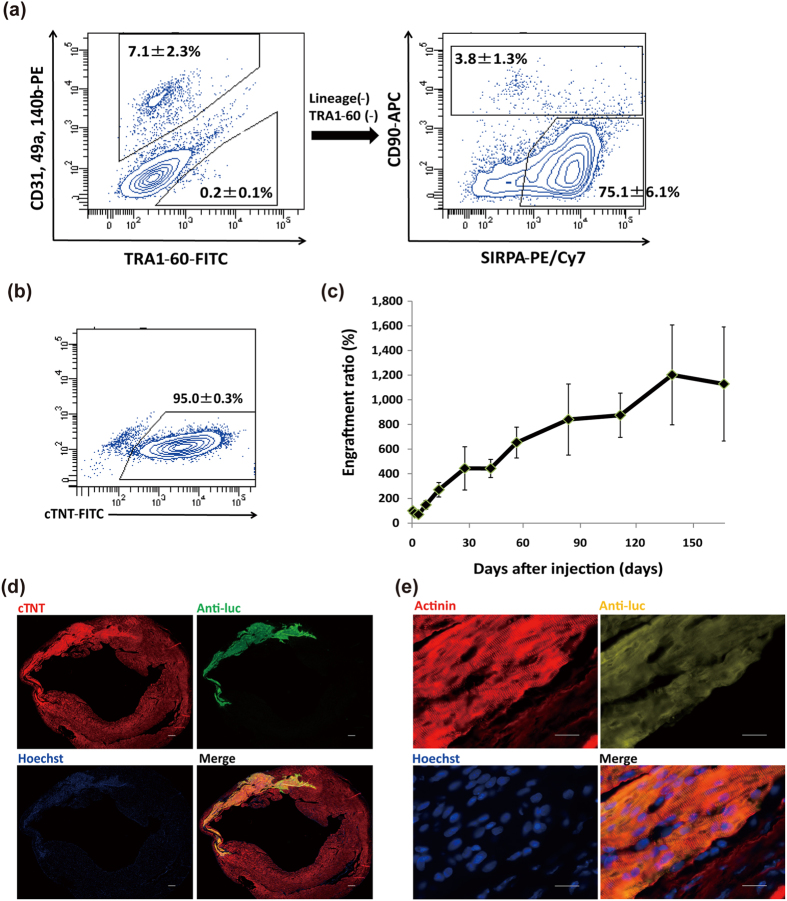
Optimized cell transplantation using another iPSC line purified by cell sorting for the cardiac surface marker, SIRPA. (**a**) Flow cytometry analysis 20 days after initial differentiation. SIPRA-positive and CD31, 49a, 90, 140b, and TRA1-60-negative cells were sorted. (**b**) Flow cytometry analysis of the sorted cells stained by cTNT. (**c**) Follow-up bioluminescence imaging of injected day20 purified-CMs. Values are mean ± SE. (**d**) Immunostaining of engrafted CMs 6 months after the initial injection of day20 CMs for cTNT (red), anti-luc (green), and Hoechst (blue). Scale bars: 300 μm. (**e**) Immunostaining of engrafted CMs at high magnification: red, actinin; yellow, anti-luc; blue, Hoechst. Scale bars: 20 μm.

To confirm whether the findings in this study depend on clones or purification methods, we investigated another iPSC line (692D2) that was generated by the integration-free episomal vector method^23^,^24^. 20 days after the initial differentiation, we purified CMs by sorting SIRPA-positive and lineage-negative cells, as previously established^25^ (Fig. 7a). After sorting, we obtained CMs with over 95% purity (Fig. 7b). Subsequently, we injected these purified CMs into infarcted heart (n = 8) and observed the engraftment by *in vivo* imaging. We found a similar time course in the luminescence signal to that of CMs from MYH6-EIP4 iPSCs (Fig. 7c). The luminescence signal at 6 months after injection was about 11-fold higher than the initial intensity. Additionally, in the histological analysis, large amounts of grafted CMs with organized sarcomere structures were observed 6 months after the injection (Fig. 7d,e). These results suggest that our findings are independent of clones or purification methods.

## Discussion

In the present study, we show that the engraft capacity of derived CMs varies with the differentiation stage. Among the several cell types examined, we found that iPSC-CMs injected 20 days after the initial differentiation (day20 CMs) were optimal for engraftment in mouse heart. Furthermore, once engrafted, they showed high graft growth with increased proliferation capacity within the first 3 months of injection, but lost their proliferation capacity and became non-proliferative, mature CMs thereafter.

We compared the grafting efficiency after transplantation of different iPSC-CM types (Fig. 2a–d). In the first few days after injection, we observed a rapid decrease in the luminescence signal, suggesting the importance of entrapment in the heart without wash out, as previously described^26^. Middle-stage iPSC-CMs (day20 and day30 CMs) were more likely to survive and engraft just after injection compared to early ones (day8 CMs). We therefore speculated that cellular conditions, such as the cell size or molecules on the cell surface, may render middle-stage iPSC-CMs more likely to attach to the host environment. In addition, the Ki67 ratio *in vitro* was higher at day 20 than at day 30, suggesting day20 CMs had better proliferation capacity (Fig. 1f). We attribute the different luminescence signal increase of the two cell types to this finding. At the same time, longer culturing, as was the case for day30 CMs, could make dissociation from the embryoid bodies difficult, which would decrease both cell viability and survival after injection.

Importantly, we observed that the growth of engrafted CMs correlated with cell-cycle activation, which means proliferation *in vivo* after transplantation. Our findings are compatible with a previous report which showed the grafts of ES cell-derived CMs grew 7-fold in graft size the first 4 weeks after injection^27^. By using bioluminescence imaging to estimate the number of engrafted cells, we managed to successfully follow the cells up to 6 months after injection, finding they proliferated well up to 1 month, gradually slowed in their proliferation over the next 2 months, and lost their proliferation capacity thereafter, instead showing structural maturation (Figs 5e,f, 6 and 7e and Supplementary Fig. S8 online). These findings are in accord with postnatal CMs losing their proliferation capacity 1 to 2 weeks after birth and then shifting to a maturation and hypertrophic phase^28^,^29^,^30^.

Because we observed different proliferation activity between *in vivo* and *in vitro* models, we investigated cell cycle-related genes, finding they were activated more in iPSC-CMs than terminally differentiated adult-CMs (Supplementary Fig. S12a,b online). Additionally, graft growth was also higher when cells were injected into acute infarcted hearts than in normal hearts. Recently, investigators reported that oxygen concentration and oxidative DNA damage affected cardiomyocyte cell cycle status and that the degree of mechanical loading was related to cardiomyocyte proliferation^31^,^32^. Additionally, several cytokines and matricellular proteins, such as bFGF, IGF1, and periostin, were reported as more activated in infarcted hearts than in normal healthy hearts^33^,^34^,^35^. IGF1 and periostin were previously reported to activate cardiomyocyte proliferation via the PI3 kinase/AKT pathway^36^,^37^. Moreover, contact with the host myocardium and microenvironment could activate the cell cycle of engrafted CMs^38^,^39^. The above reasons could have influenced graft growth and may be important factors when optimizing iPSC-CMs proliferation.

We observed that optimized cell therapy had sustained therapeutic effects on heart function during 12 weeks after injection. However, other reports demonstrated only transient improvement of heart function^20^,^40^. By optimizing the injected cells, we achieved massive engraftment of iPSC-CMs that could replace the scar areas into viable myocardium. Furthermore, we observed that a substantial amount of host CMs survived in the infarcted area of grafted hearts compared to control hearts, in which the LV wall changed entirely into thin scar tissue (Figs 3d,e and 5c,d, and Supplementary Fig. S9 online). One possibility is that grafted cells attenuated loss of host CMs, resulting in the sustained improvement of cardiac function, whereas it was not obvious that engrafted CMs functioned as working myocardium via direct remuscularization.

Through our optimization process, we found the highest engraftment ratio using day20 CMs. However, this ratio may be partially attributable to our use of NOG mice as recipients, since these mice are more immune deficient than other common mouse models. Therefore, when extending our findings to auto- and allo-transplantation, how the immune system affects the ER should be considered^41^,^42^. In addition, although we investigated two iPSC lines with different purification protocols, different methods for the differentiation and preparation of graft cells may influence the cell state and thus contribute to different engraft efficiencies.

There are several limitations in this study. First, we used mice as our platform for cell therapy. Mice display rapid heart rates, approximately 600 beats per minutes, which is much faster than what human cardiomyocytes can maintain. The significant difference in heart rate could compromise an accurate assessment of any pro- or anti-arrhythmic effects by the transplanted cells. In addition, there was no evidence of electromechanical coupling of engrafted CMs with host CMs. Further experiments using larger animal models that have heart rates closer to those of humans are essential to elucidate whether our optimized conditions increase or decrease the risk of arrhythmia in damaged heart. Second, the failure of 1:1 host-graft coupling due to the different heart rates hinders the evaluation of the effect of transplanted CM contraction on cardiac pump function. Importantly, though we evaluated engraftment efficacy of iPSC-CMs at several differentiation stages, it does not outright establish that day20 CMs have the best therapeutic potential to treat damaged hearts. Several studies have suggested that cell therapy using cardiomyocytes have beneficial effects by paracrine effects, not by direct remascularization^43^. If this is the case, large engraftment does not necessarily lead to efficient therapeutic benefits. Additionally, other cell types, such as endothelial cells and bone marrow cells, also have been reported to improve cardiac function after myocardial infarction via the formation of new blood vessels, paracrine effects, or other mechanisms^12^. Therefore, more detailed comparisons of CMs and other cell types are needed to identify the best cell type for cardiac cell therapy. Finally, although we directly injected cells into the heart, other methods for transplantation, such as coronary artery injection and the cell sheet method, also deserve consideration^3^,^4^,^5^,^44^,^45^. The optimal conditions for cells using these strategies may be different from those reported here and deserve further study.

In conclusion, we have identified an optimal differentiation stage for high engraftment in cardiac cell therapy. This efficient transplantation model enabled us to dissect the fate of transplanted CMs, as we observed the transition from the proliferative phase to maturation phase in host hearts. We expect these results to advance the potential clinical application of iPSC-CMs in cardiac cell therapy.

## Methods

All of the experimental protocols were approved by the Kyoto University Animal Experimentation Committee, and the methods were performed in accordance with the Guidelines for Animal Experiments of Kyoto University and the Guide for the Care and Use of Laboratory Animals by the Institute of Animal Resources.

### *In vitro* iPSC cardiac differentiation

The human iPSC line 201B7, established using the four Yamanaka factors, was used^6^. Additionally, we used integration-free iPSCs, established by episomal vector^23^. iPSCs were maintained on SNL feeder layers in primate ES cell medium (ReproCell, Japan) supplemented with 4 ng/ml bFGF (Wako, Japan). iPSC-CMs were generated using an embryoid body (EB) method as previously described with modifications^14^,^46^. In brief, undifferentiated iPSCs were detached and dissociated into single cells by 5-min incubation with Accumax (Innovative Cell Technologies, Inc, San Diego, CA). The cells were then suspended in StemPro34 medium supplemented with 2 mM L-glutamine, 50 μg/ml ascorbic acid, 4 × 10^-4^ M monothioglycerol, 150 μg/ml transferrin, 10 μM Y-27632 (WAKO) and 2 ng/ml human recombinant BMP4 (R&D Systems, Minneapolis, MN) and allowed to form EBs by being placed into a low-attachment 96-well dish and cultivated for 24 hours. On day 1, media, which included human recombinant activin A (R&D Systems), BMP4, and bFGF (R&D Systems), were added into the wells. Final concentrations were as follows: activin A, 6 ng/ml; BMP4, 10 ng/ml; and bFGF, 5 ng/ml. On day 3, EBs were collected from each well and dissociated into single cells by 5-min incubation with Accumax. The cells were then suspended in differentiation media supplemented with 10 ng/ml VEGF (R&D Systems) and 1 µM IWP-3, a Wnt inhibitor (Stemgent, Cambridge, MA). Approximately 20000 cells were then put in each well to form aggregates for 4 days. On day 7, the media were changed to StemPro 34 media supplemented with 2 mM L-glutamine, 50 μg/ml ascorbic acid, 4 × 10-4 M monothioglycerol, 150 μg/ml transferrin, 10 ng/ml VEGF and 5 ng/ml bFGF. For the maintenance of iPSC-CMs, the culture media were refed every 2–3 days. On the day of cell injection, EBs were dissociated by collagenase I for 3–6 hours and Accumax for 10 minutes. The dissociated cells were then subjected to cell sorting (Ariall, BD Bioscience, San Jose, CA). Cell number was counted by TC20 automated cell counter (Bio-Rad, Hercules, CA).

### Generating cardiac specific MYH6-GFP reporter cell lines

Cardiomyocyte-specific EGFP reporter lines were generated using the piggyBac transposon system, as previously described^47^,^48^. A sequence containing the human MYH6 promoter (−4391 ~ +1051) was cloned, and an EGFP-Ires-puromycin cassette was inserted downstream of this sequence. At the 3′ end of the construct, a phosphoglycerate kinase promoter-driven neomycin resistance cassette flanked by loxP-sites was inserted. 201B7 iPSCs were prepared for transfection as follows. After dissociating iPSCs into single cells, 1 μg MYH6-GFP plasmid and 1 μg PBase plasmid were transfected into iPSCs with FuGENE HD (Roche, Indianapolis, IN). Forty-eight hours after transfection, neomycin was added to select transfected cells. We subcloned the transfected cell lines and confirmed these cells expressed EGFP after undergoing cardiomyocytes differentiation and that 97.1% of the sorted EGFP-positive cells were cardiomyocytes positive for cTNT (Supplementary Fig. S1 online).

### Gene expression microarray analysis

For gene expression analysis, total RNA was extracted by the miRNeasy Kit (Qiagen, Valencia, CA). We used 3 fetal heart samples (16, 20, 24 weeks) and 3 adult heart samples as a control (BioChain Institute Inc. Newark, CA). Microarray analysis was conducted using the SurePrint G3 Human Gene Expression 8 × 60K Kit (G4851A; Agilent technology, Palo Alto, CA) with the Microarray Scanner System (G2565CA; Agilent technology). Data were analyzed using GeneSpring (Agilent Technology). Microarray data are available at the Gene Expression Omnibus (http://www.ncbi.nlm.nih.gov/geo/) under accession no. GSE60634.

### *In vivo* bioluminescence imaging

To monitor cell engraftment, we generated cell lines that continuously expressed luciferase. These lines were generated by the introduction of a CAG promoter-driven luciferase-expressing cassette to the MYH6-GFP reporter line (MYH6-EIP4) and the integration-free iPSC line (692D2) using the piggyBac transposon system^47^,^48^. For bioluminescence imaging, mice were anesthetized by isoflurane, and D-luciferin (SPI, Japan) was administered at a dose of 200 mg/kg i.p. Images of mice were captured by an *in vivo* bioluminescence imaging system (IVIS, Caliper Life Sciences, Hopkinton, MA). Follow-up imaging was conducted just after cell injection and on days 1, 3, and 7 and weeks 2, 4, 8, and 12 after the initial injection day. In long-term follow-up, additional images were captured on weeks 16, 20, and 24 after the initial day. The engraftment ratio (ER) was calculated by the luminescence signal at each time point divided by the signal just after injection on day 0.

### Surgical procedures

Male non-obese diabetic/severe combined immunodeficiency NOG mice (8–10 weeks old) were intubated with a 20-gauge angiocath (TERUMO, Japan) and mechanically ventilated under general anesthesia with 2% isoflurane. The heart was exposed by left anterolateral thoracotomy. Injections into the healthy hearts of NOG mice were made at a single site of the LV free wall with a total volume of 20 μl IMDM (Life Technologies, Gaithersburg, MD) supplemented with 10 μM Y-27632 containing 2.5 × 10^5^ cells. Injection procedures were performed by a Hamilton syringe with a 30-gauge needle. For injections into damaged hearts, myocardial infarctions were generated by ligation of the left anterior descending artery using a 8-0 Prolene suture (Ethicon, Inc., Johnson and Johnson, Somerville, NJ). Myocardial infarction was confirmed by visual inspection of myocardial blanching. In treated mice, 1.0 × 10^6^ purified iPSC-CMs were injected at 2 sites of the infarcted areas with 10 μl IMDM and 10 μM Y-27632. IMDM alone with 10 μM Y-27632 was injected into control mice.

### Echocardiography

Before surgery and every two weeks thereafter, animals were anesthetized mildly with inhaled isoflurane (Abbott Japan, Japan) and their cardiac function, including left-ventricular diastolic/systolic dimension (LVDd/Ds) and fractional shortening (FS), was measured using transthoracic echocardiography (GE Vivid S, GE, Milwaukee, IL). FS was calculated as 100 × (LVDd-LVDs)/LVDd (%).

### Histological analysis

For standard immune-fluorescence analysis, all hearts were fixed with 4% paraformaldehyde (PFA) for 12 hours at 4 ^o^C and were replaced in 15% sucrose for 3 hours followed by 30% sucrose for 12 hours at 4 ^o^C. These samples were embedded in an optical cutting temperature (OCT) compound (Tissue Tek) and frozen on dry ice. Five-micron sections were stained. Immunostaining was performed with antibodies against cTNI (Santa Cruz, Santa Cruz, CA), cTNT (Thermo Scientific), human nuclei (Millipore, Bedford, MA), anti-luciferase (anti-luc) (Promega), and human NKX2.5 (R&D Systems). Nuclear staining was performed by Hoechst33342 (Life technologies), and cell proliferation was assessed by immunostaining with antibody against Ki67 (Cell Signaling Technology, Beverly, MA). Masson trichrome staining was performed according to standard procedures at Center for Anatomical, Pathological, and Forensic Medical Researches, Kyoto University. Wheat germ agglutinin staining (Life technologies) was performed to measure the cross-sectional area of cardiomyocytes. In this staining, grafted CMs were identified by staining against cTNT (Abcam), which did not cross-react with mouse. Tissues were visualized using a fluorescence microscope (BZ9000 and BZ-X700, Keyence, Japan). Images of the sarcomere maturation process were visualized by confocal imaging (FV1000, Olympus, Japan) immunostained with antibody against α-actinin (Sigma) and anti-luc. For correlative light and electron microscopy, fixed hearts were cross-sectioned into 100-μm-thick slices using a vibrating microtome (VT 1000S, Leica), rinsed in PBS for 3 × 15 min, blocked in a blocking buffer (PBS containing 5% normal donkey serum, 1% bovine serum albumin, 1% cold water fish gelatin, 20 mM glycine, and 0.1% saponin) for 2 hours on ice, and incubated with an anti-luc antibody (Promega) overnight at 4 °C. After washing in a 10x-diluted blocking buffer 5 times (15 min each time), the slices were incubated with an anti-donkey antibody conjugated with Alexa 594 for 4–6 hours at 4 °C and washed again. The immuno-stained slices were imaged on a laser-scanning microscope (FluoView FV1000, Olympus) using a 10x objective (NA 1.42) to identify engrafted iPSC-CMs, followed by washing in 0.15 M sodium cacodylate buffer (pH7.4) and fixation in 2%PFA and 2% glutaraldehyde in 0.15 M sodium cacodylate for 3 hours at 4 °C. The post-fixed slices were further stained in 2% osmium tetroxide with 0.8% potassium ferrocyanide and then in 1% uranyl acetate at 4 °C, washed with water, dehydrated, and embedded in Durcupan ACM resin (Sigma-Aldrich) as described previously^49^. Clumps of iPSC-CMs were located in 80-nm-thick sections correlating to immuno-fluorescence images with the assistance of anatomical landmarks including blood vessels and papillary muscles and imaged on a Tecnai Sprit microscopy (FEI) equipped with a 2k x 2k CCD camera operating at 120 kV.

## Statistical Analysis

Statistical analysis was performed by commercially available software (GraphPad Prism, GraphPad Software, San Diego, CA). Data were analyzed by an unpaired t test or one-way ANOVA with Tukey’s posthoc test. Differences were considered statistically significant at p < 0.05.

## Additional Information

**How to cite this article**: Funakoshi, S. *et al.* Enhanced engraftment, proliferation, and therapeutic potential in heart using optimized human iPSC-derived cardiomyocytes. *Sci. Rep.* **6**, 19111; doi: 10.1038/srep19111 (2016).

## Supplementary Material

Supplementary Information
