# Supplementary material for: Enhanced engraftment, proliferation, and therapeutic potential in heart using optimized human iPSC-derived cardiomyocytes
Source: Sci Rep. 2016 Jan 8;6:19111. doi: 10.1038/srep19111 (PMC4705488; doi:10.1038/srep19111)
Supplement: Supplementary Information [file srep19111-s1.pdf]

Enhanced engraftment, proliferation, and therapeutic potential in heart using optimized human iPSC-derived cardiomyocytes

Shunsuke Funakoshi[1,2], Kenji Miki[1], Tadashi Takaki[1], Chikako Okubo[1], Takeshi Hatani[1,2], Kazuhisa Chonabayashi[1], Misato Nishikawa[1], Ikue Takei[1], Akiko Oishi[1], Megumi Narita[1], Masahiko Hoshijima[4], Takeshi Kimura[2], Shinya Yamanaka[1,3], Yoshinori Yoshida[1]

[1]Center for iPS Cell Research and Application, Kyoto University, Kyoto, Japan. [2]Department of Cardiovascular Medicine, Kyoto University Hospital, Kyoto, Japan. [3]Gladstone Institute of Cardiovascular Disease, San Francisco, USA. [4]Center for Research in Biological Systems and Department of Medicine, University of California at San Diego, La Jolla, California, USA

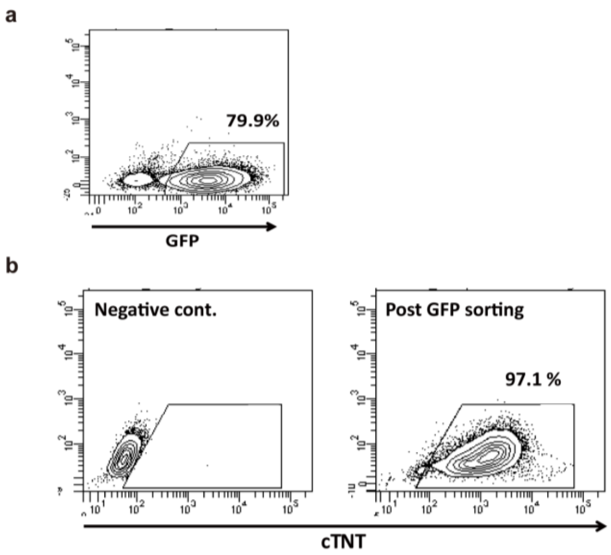

Supplementary Figure 1: Flow cytometry analysis of differentiated CMs in vitro. (**a**) Flow cytometry showed that about 80% of day20 CMs had differentiated into GFP-positive CMs in vitro. (**b**) Flow cytometry analysis of sorted GFP-positive cells. Left panel: unstained control; right panel: antibody stains against cTNT.

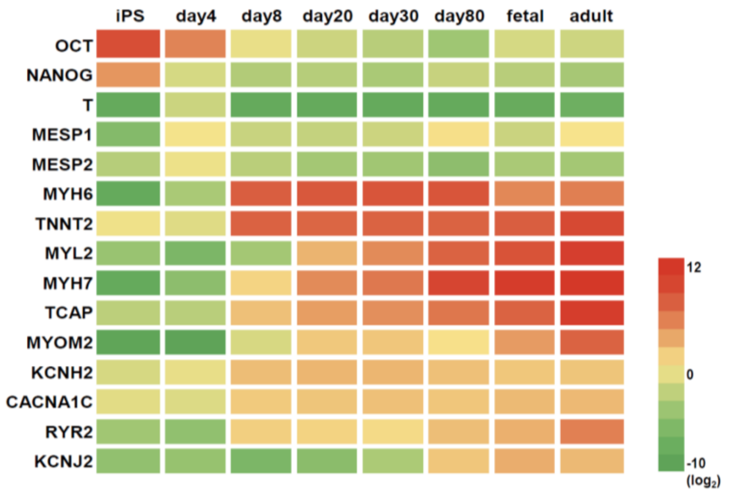

Supplementary Figure 2: Gene expressions during differentiation. Relative expression of pluripotent genes, mesodermal genes, and cardiac genes are shown. Sample numbers of cells at each stage are as follows: iPSC, n=1; day4 cells, n=1; purified day8, 20, and 30 CMs, n=3 each; purified day80 CMs, n=1; fetal heart and adult heart, n=3 each.

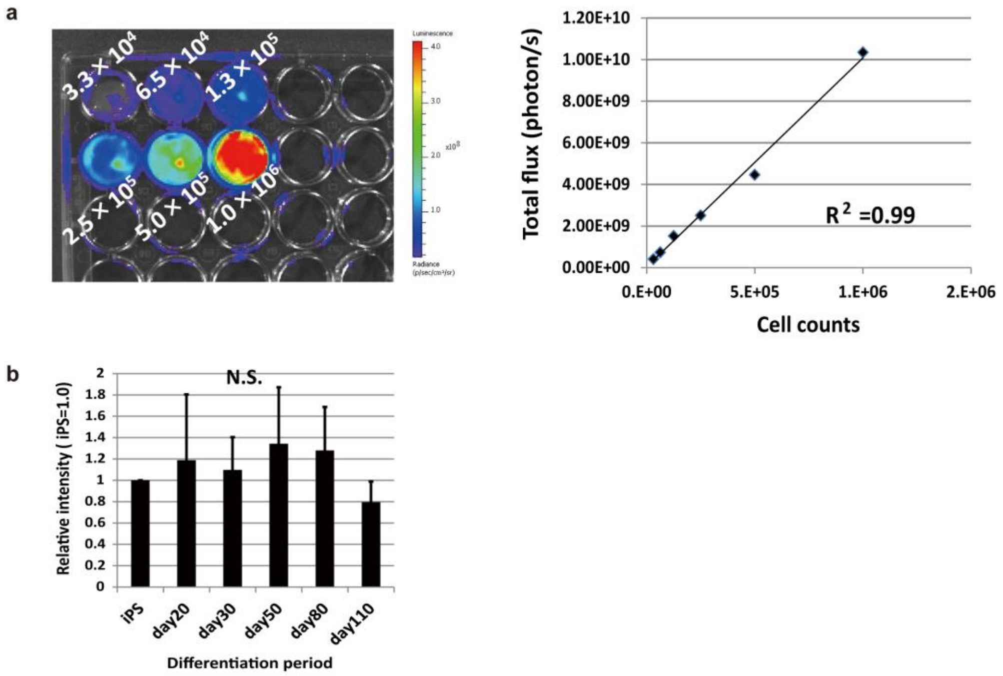

Supplementary Figure 3: Bioluminescence signals correlated with cell numbers. (**a**) Correlation of the luminescence signal intensity and cell number in vitro (left). R^2 equals the coefficient of determination (right). (**b**) Signal intensity during differentiation in vitro. A value of 1 indicates the signal intensity of iPSCs in the undifferentiated state. There were no significant changes in signal intensity during differentiation in vitro by one-way ANOVA (n=3 each). Values are mean±SD.

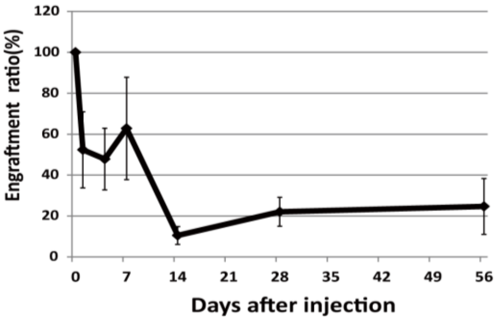

Supplementary Figure 4: Follow-up bioluminescence imaging of injected GFP-negative non-CMs. Imaging after injection of $2.5x10^5$ GFP-negative non-CMs showed no increase in signals during the observation period. Values are mean±SE.

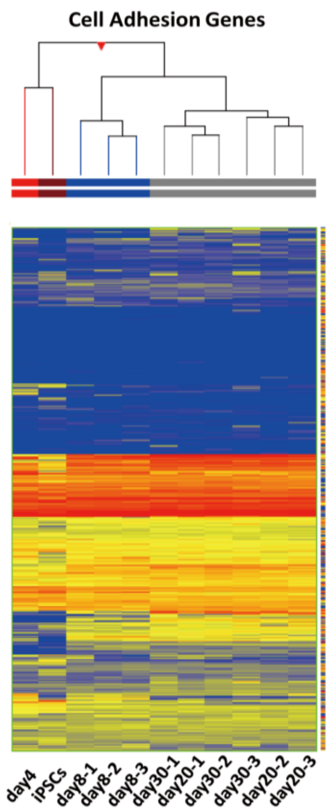

Supplementary Figure 5: Clustering analysis in cell adhesion genes among samples from iPSCs to day30 CMs.

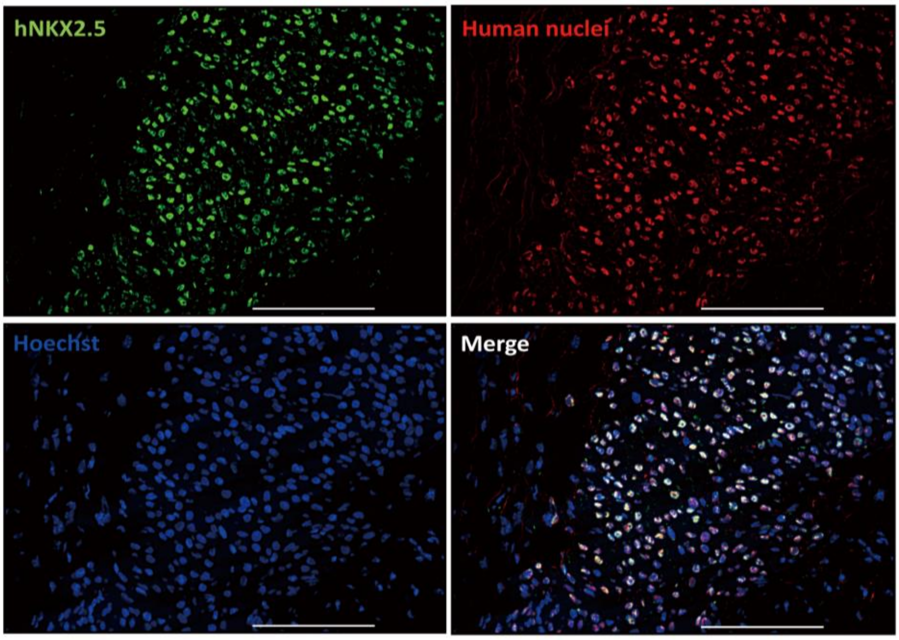

Supplementary Figure 6: Most engrafted cells were CMs that expressed cardiac NKX2.5. Immunostaining of engrafted CMs for human NKX2.5 (green), human nuclei (red), and Hoechst (blue). Scale bars: 100 μm.

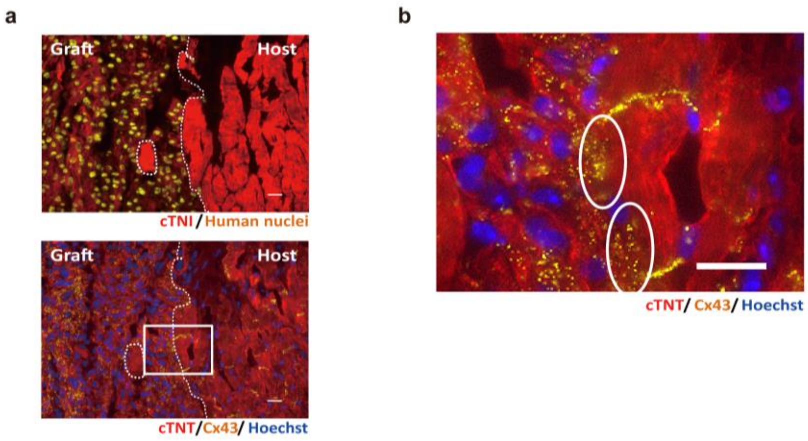

Supplementary Figure 7: Connexin 43 (Cx43) expression between host and graft CMs. (**a**) Upper: Immunohistochemistry of engrafted CMs 3 months after the initial injection of day20 CMs for human nuclei (yellow) and cTNI (red). Lower: Serial section of the upper image. Immunohistochemistry for Cx43 (yellow), cTNT (red), and Hoechst (blue). The white dotted lines show the borderline between the host and grafts. Scale bars: 20 μm. (**b**) Magnified image of the white box in (**a**). The white circle shows Cx43 between the grafted CMs and host CMs. Scale bar: 20 μm.

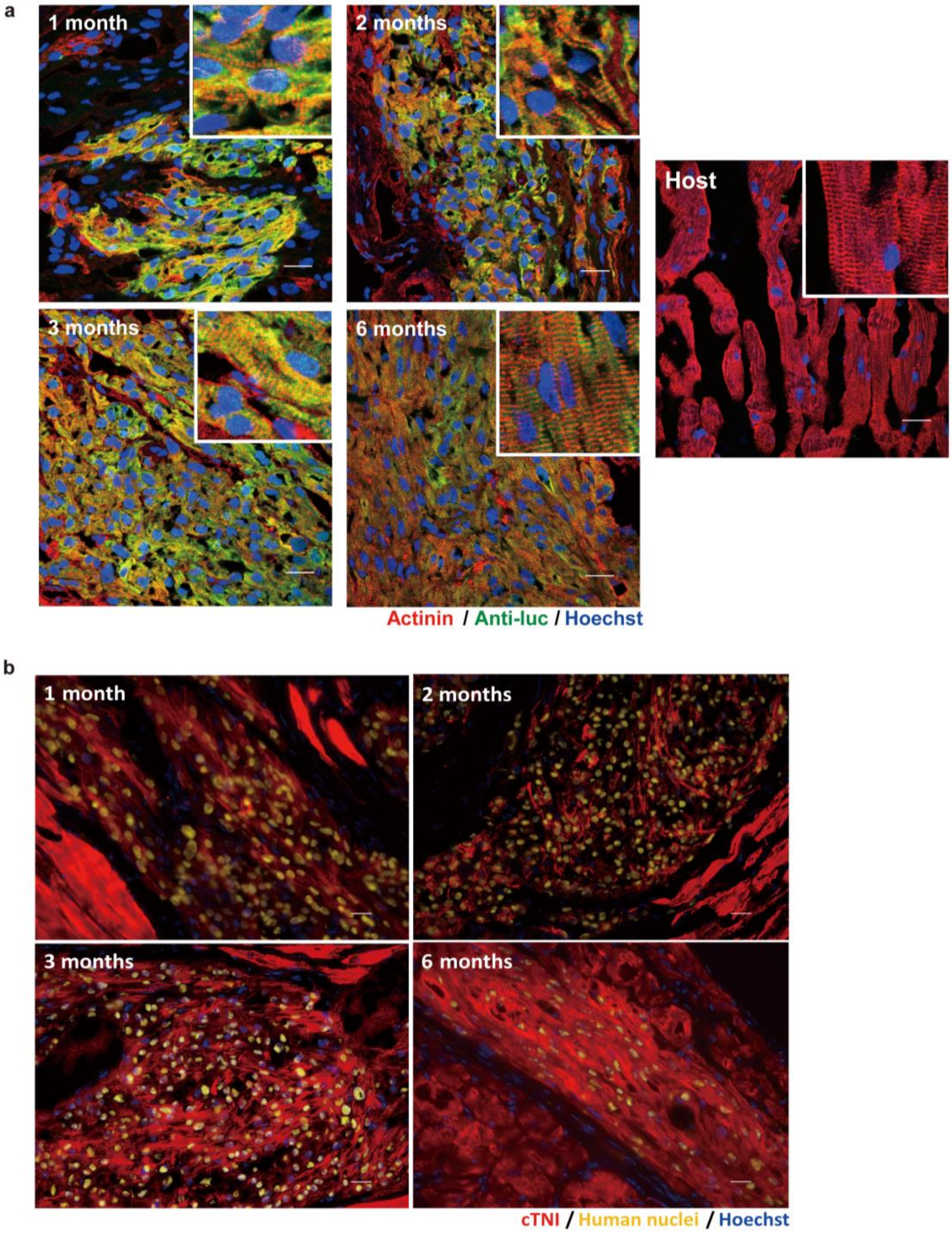

Supplementary Figure 8: Time course of sarcomere maturation in vivo shows a gradual maturation process of sarcomeric structures in engrafted CMs. (**a**) Time course of structural maturation after engraftment. Immunostaining for actinin (red), anti-luc (green), and Hoechst (blue). Scale bars: 20 μm. (**b**) Time course of cTNI expression in engrafted CMs. Immunostaining for cTNI (red), human nuclei

(yellow), and Hoechst (blue). Scale bars: 20 μm.

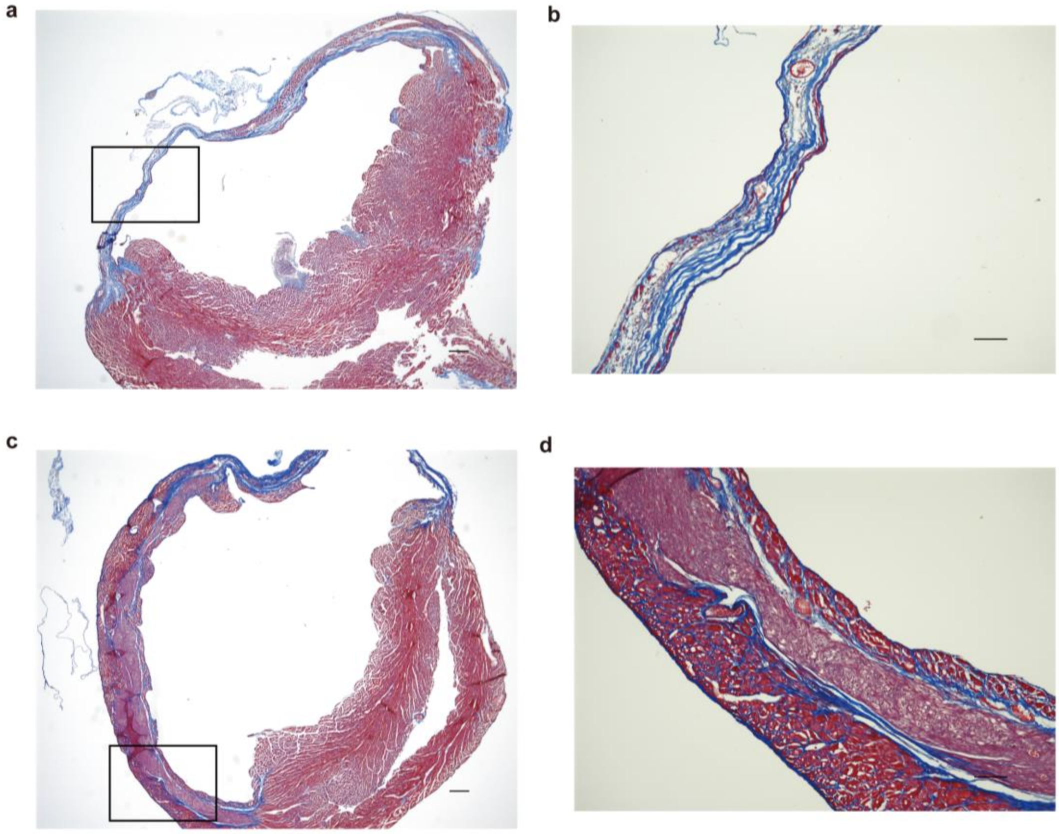

Supplementary Figure 9: Trichrome-staining of treated heart 6 months after injection. (**a**) Trichrome-staining of control heart treated by medium only. Scale bar: 300 µm. (**b**) Magnified image of the black box in (**a**). Scale bar: 100 µm. (**c**) Trichrome-staining of iPSC-CMs treated heart. This image was obtained by serial section from the same heart seen in Fig. 5C. Scale bar: 300 µm. (**d**) Magnified image of the black box in (**c**). This image is the same area seen in Fig. 5d. Scale bar: 100 µm.

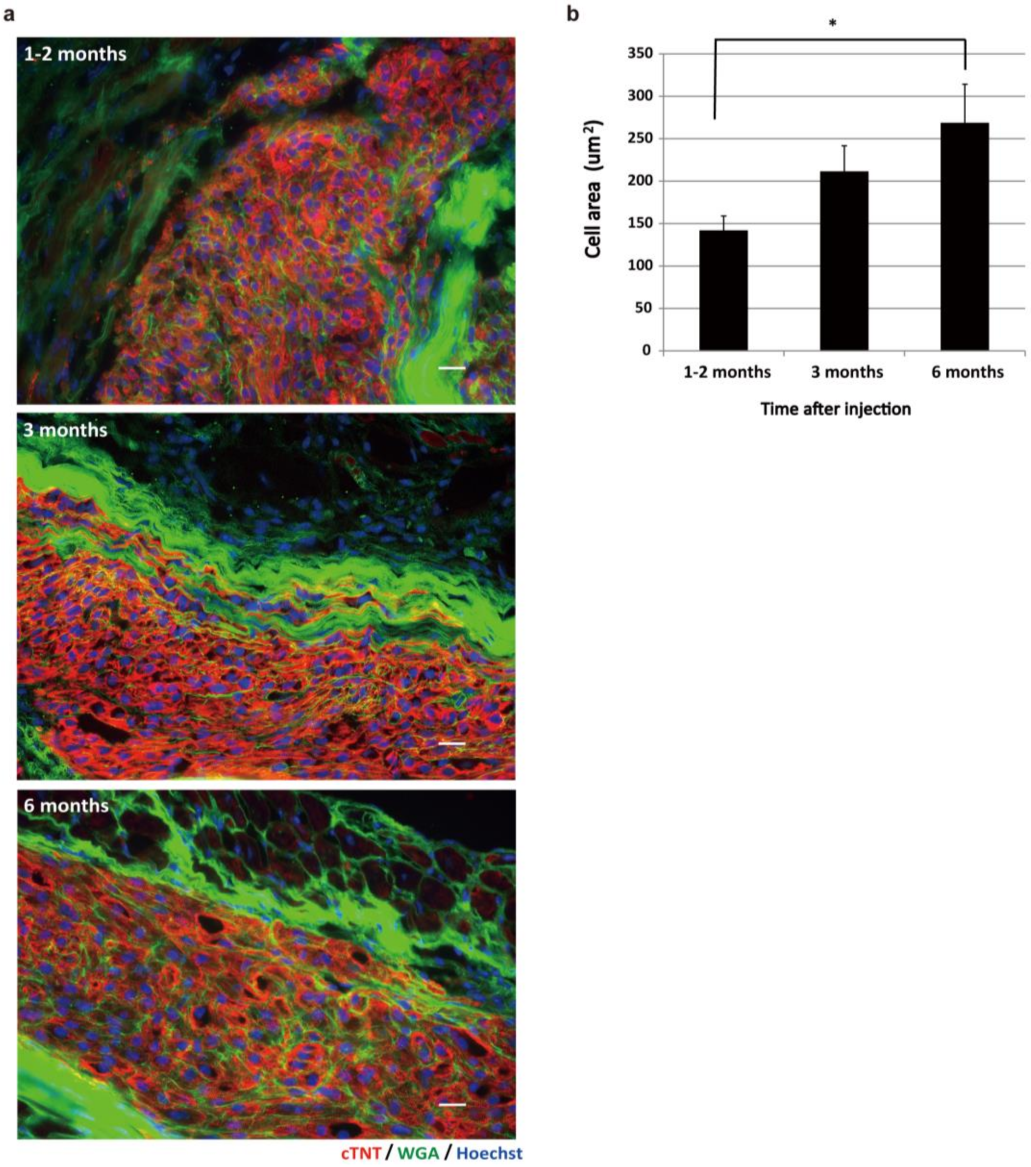

Supplementary Figure 10: Measurement of engrafted cardiomyocyte size. (a) Representative images of engrafted CMs 1-2 months, 3 months, and 6 months after injection. Wheat germ agglutinin (WGA) staining (green), immunostaining for cTNT (red), and Hoechst (blue). This antibody against cTNT did not cross-react with mouse CMs, which means cTNT-positive lesions were grafted areas. Scale bars: 20 μm. (b) Comparison of cardiomyocyte cell areas at several time points. Values are mean±SD. *p<0.05 by one-way ANOVA followed by

Tukey’s posthoc test.

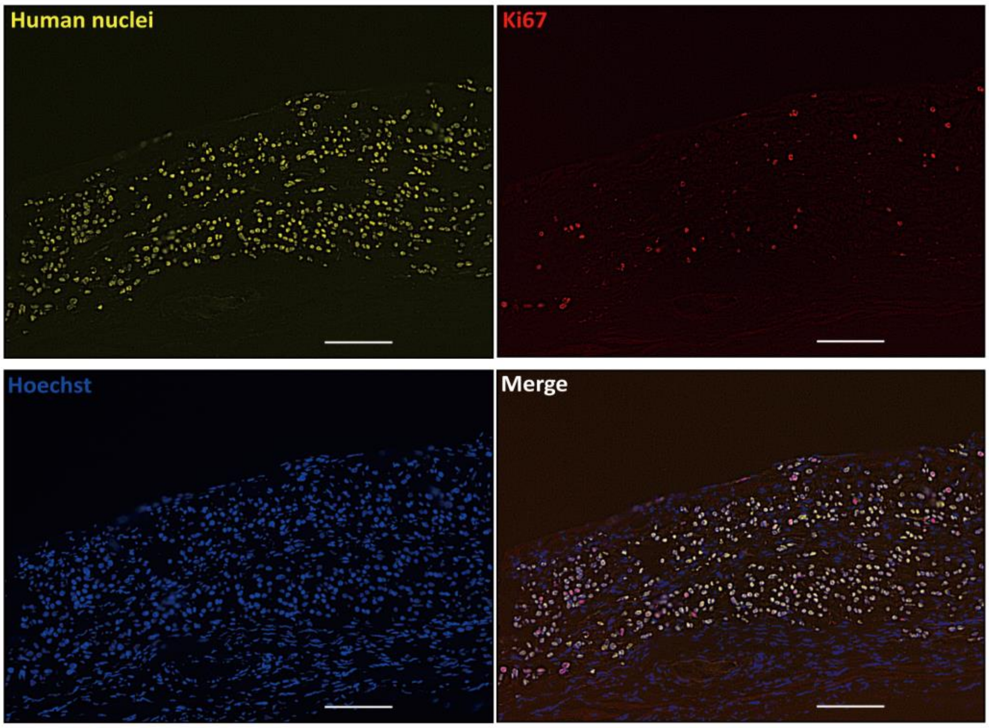

Supplementary Figure 11: Immunohistochemistry of engrafted CMs 1 month after the initial injection of day20 CMs for human nuclei (yellow), Ki67 (red), and Hoechst (blue). Scale bars: 100 μm.

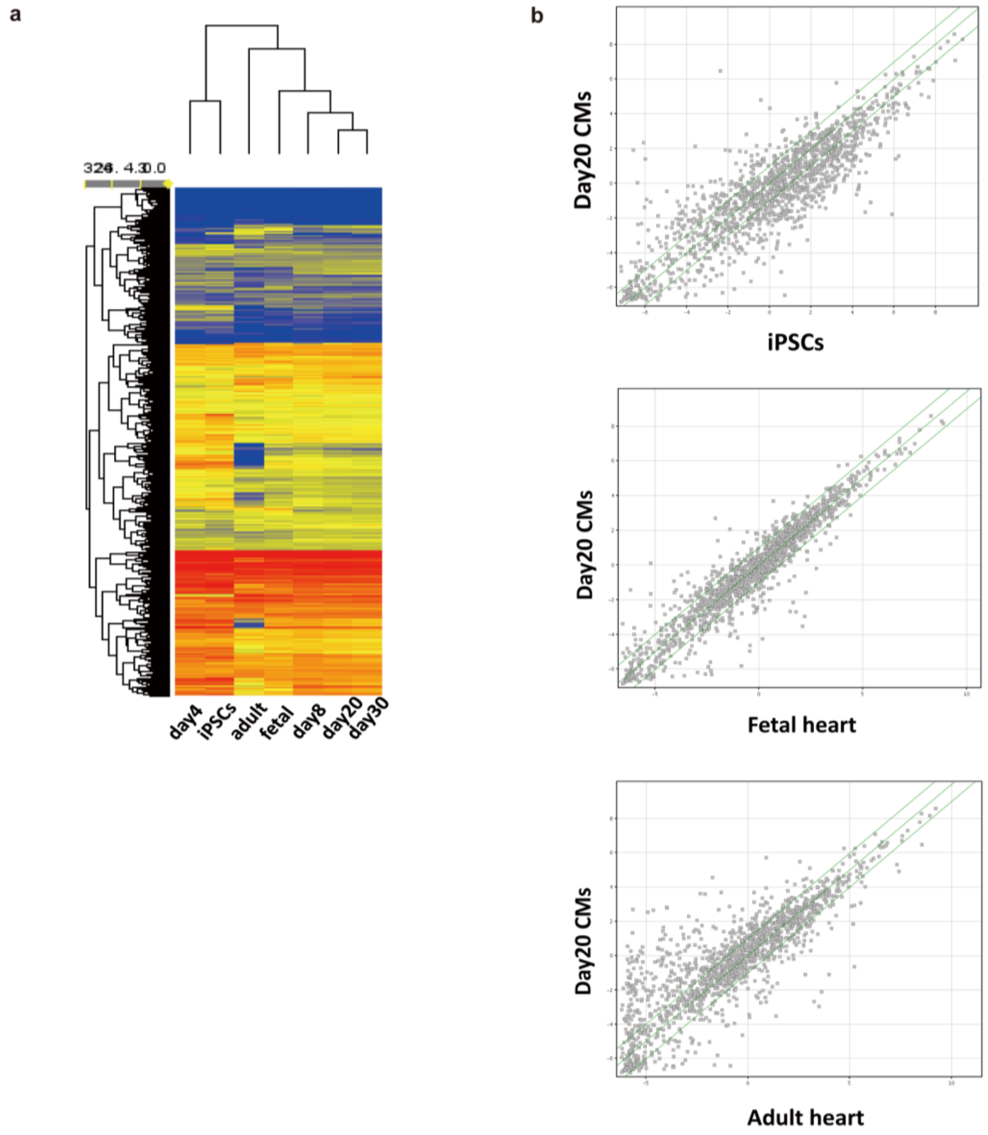

Supplementary Figure 12: Expressions of cell cycle-related genes in iPSC-CMs. (**a**) Hierarchical clustering of the cell cycle-related gene expression data obtained from iPSCs, day4 mesodermal cells, and purified day8, day20, and day30 CMs. iPSC-CMs were located in clusters close to fetal heart samples. (**b**) Scatter plots of the expression of cell cycle-related genes: day20 CMs vs. iPSCs, fetal heart, and adult heart. Sample numbers are as follows: iPSC, n=1; purified day20 CMs, n=3; fetal heart, n=3; adult heart, n=3.

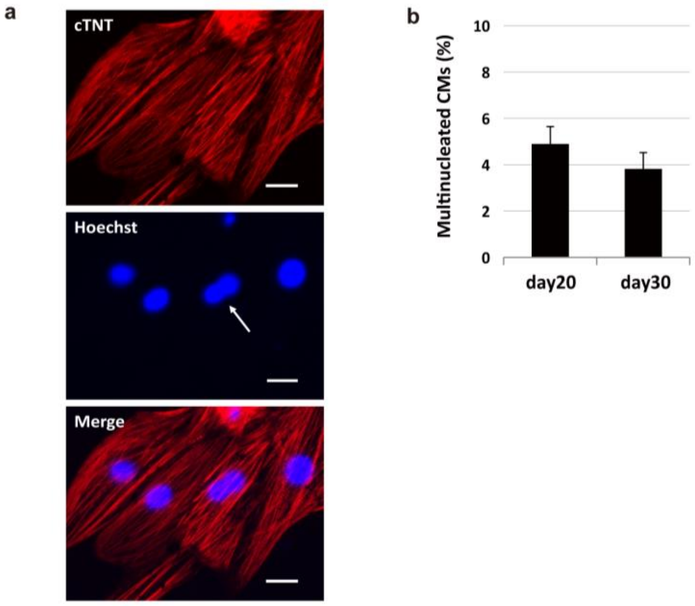

Supplementary Figure 13: Multinucleation of differentiated iPSC-CMs. (a) Immunohistochemistry of day30 CMs for cTNT (red) and hoechst (blue). The white arrow marks a multinucleated CM. Scale bars: 20 μm. (b) The ratio of multinucleated CMs at day20 and day30 (n=3 each). Values are mean±SD.
